# Risk Factors for Unplanned Early Implantable Port Catheter Removal in Adult Leukemia/Lymphoma Patients: Cancer Type or Different Degrees of Cytopenia?

**DOI:** 10.3390/cancers17091505

**Published:** 2025-04-29

**Authors:** Ming-Shian Lu, Chih-Chen Chen, Che-Chia Chang, Chien-Chao Lin, Ching-Chuan Hsieh

**Affiliations:** 1 Department of Surgery, Chang Gung Memorial Hospital at Chiayi, Puzi City 61363, Taiwan; cs6063@cgmh.org.tw (M.-S.L.); m0686@cgmh.org.tw (C.-C.C.); m7018@cgmh.org.tw (C.-C.L.); 2 Department of Medicine, College of Medicine, Chang Gung University, Taoyuan 33302, Taiwan; 3 Department of Medicine, Chang Gung Memorial Hospital at Chiayi, Puzi City 61363, Taiwan

**Keywords:** lymphoma, leukemia, port A, complications, removal, scored patient-generated subjective global assessment

## Abstract

(1) Background: Implantable port catheters are vital for cancer treatment, but complications such as infections and mechanical failures pose challenges. Lymphoma and leukemia patients’ unique cellular abnormalities may influence these risks. This study aimed to determine whether the underlying disease or varying degrees of cytopenia increase the risk of unplanned early port removal. (2) Methods: We conducted a single institution retrospective study that included 368 patients with lymphoma or leukemia who received implantable venous access ports between January 2015 and December 2022. Propensity score matching was employed to compare patients with and without early removals. (3) Results: Univariate analysis revealed statistically significant differences between early and non-early port removal for cancer, hemoglobin, and PG-SGA scores. Cox proportional hazard analysis demonstrated that leukemia patients exhibited a 4.5 times higher risk for unplanned early catheter removal than lymphoma patients did (HR 4.589, 95% CI 1.377–15.299, *p* = 0.013), while patients with normal nutrition, based on the PS-SGA, demonstrated a 75% lower risk of unplanned early catheter removal than those with any degree of malnutrition did (HR 0.258, 95% CI 0.116–0575, *p* < 0.001). Unplanned early catheter removal negatively impacted patient survival. (4) Conclusions: The type of cancer, rather than individual cytopenias, is an independent factor influencing clinical outcomes in lymphoma and leukemia patients.

## Simple Summary

Port implantation is a simple surgical procedure typically done under local anesthesia. These vascular ports are designed to provide repeated vascular access for patients requiring intravenous fluids, drug delivery or blood transfusions. In hematologic cancers such as lymphoma and leukemia, distinct blood cells characteristics help determine whether the disease itself or varying types of cytopenia contribute to the risk of early port infection. Addressing this challenge is essential, as it can significantly influence patient outcomes. This highlights the importance of strategies aimed at preventing complications and optimizing patient care.

## 1. Introduction

Totally implantable port systems were introduced in the early 1980s [1] and consistently provide secure vascular access for administering chemotherapy, fluids, medications, blood products, and parenteral nutrition solutions. Infection is the primary cause of premature port removal in adult cancer patients, followed by mechanical complications and thrombosis [2,3]. The reported infection rate ranges from 0.067 to 1.2 per 1000 catheter days in oncology patients [4,5]. Patients requiring port implantation exhibit compromised immune function due to their underlying disease or associated treatments. Previous studies on implantable vascular ports for hematology patients are limited in the literature. Five studies are cited [4,6,7,8,9], showing mixed results regarding infection risk. Some indicated an increased risk, while others did not. None of these studies evaluated the impact of individual cytopenia or cancer type on infection risk. In a study involving 1747 implantable venous access ports, with 144 ports in patients with malignancies, Shim et al. reported an adjusted odds ratio of infection of 7.70 in patients with hematologic malignancies [4]. Mollee et al. reported that the type of central venous access device not only influences the risk of infection, but also that hematologic malignancies present a higher risk of infection compared to solid cancers such as esophageal or colorectal cancers [6]. In a retrospective study from three centers, Skummer et al. reported a hazard ratio of 2.61 for early port infection in patients with hematologic malignancies [7]. In contrast, Viana Taveira et al. observed no increased risk for blood stream infection in patients with hematology malignancies compared to solid cancers [8]. In a study of nearly 6000 patients, Nezami et al. reported a higher infection rate for hematology malignancy on univariate analysis, but without statistical significance on multivariate analysis [9].

Lymphoma and leukemia are two major types of hematology malignancies that usually present with different cytopenia profiles at diagnosis. Whether the disease or the cytopenia patterns influence unplanned catheter removal risk is unknown. Unplanned early removal of implantable catheters disrupts treatment schedules and delays therapy; thus, identifying risk factors associated with this condition enables clinicians to address them promptly and prevent unnecessary surgical procedures. Most reports define unplanned early catheter removal as occurring within 30 days post-implantation. Given that most treatment schedules extend beyond 30 days, we extended this period to 90 days post-implantation. This study was conducted to assess factors associated with unplanned early port removal using propensity score matching in patients with lymphoma and leukemia. The aim was to determine whether individual cytopenia or the type of cancer influenced the survival of the implanted port catheter.

This manuscript is organized into five main sections. The Introduction provides background on the clinical relevance of port catheter complications in hematologic malignancies and outlines this study’s rationale. The material and methods Section 2 details this study’s design, patient population, statistical approach, and surgical procedure. The results Section 3 presents findings from univariate and multivariate analyses, including propensity score matching, risk factor assessment, and survival outcomes. The discussion Section 4 interprets these results in the context of the existing literature, exploring the implications of cancer type and nutritional status on catheter outcomes. Finally, the conclusion Section 5 summarizes key findings and highlights the need for further research to mitigate risks of unplanned early port removal in this patient population.

## 2. Materials and Methods

### 2.1. Study Population

We recruited patients over 18 years old with lymphoma and leukemia from Chang Gung Memorial Hospital, Chiayi, who underwent a permanent implantable port procedure between January 2015 and December 2022. This retrospective study was conducted in accordance with the Declaration of Helsinki, approved by the Institutional Review Board of Chang Gung Memorial Hospital, and waived individual consent due to de-identification.

Medical records collected at the time of catheter implantation included data on age, sex, body mass index, smoking status, cancer type (leukemia or lymphoma), Charlson comorbidity index [10], laboratory values (CBC/DC, albumin level, and creatinine), catheter implantation date, catheter removal date, and the reason for catheter removal. A trained onco-nutritionist assessed the nutritional status of all cancer patients within 72 h of admission using Scored Patient-Generated Subjective Global Assessment (PG-SGA) scores [11]. The PG-SGA form encompasses four patient-generated components: weight history, food intake, symptoms, and activities and function. Additionally, it includes a professional section covering weight loss scoring, diagnosis, metabolic stress, and physical examination, leading to a total numerical score, global assessment, and nutritional triage recommendation. The PG-SGA form can be accessed at https://pt-global.org/ (accessed on 5 March 2025). Based on their PG-SGA scores, patients were classified as having normal nutrition (score 0–1), mild malnutrition (score 2–3), moderate malnutrition (score 4–8), or severe malnutrition (score > 9). We defined catheter survival as the duration from the catheter implantation date to the catheter removal date (last follow-up or death if not removed). We reviewed all medical records until the removal of the implantable catheter, patient loss to follow-up, or the patient’s death. The cancer case manager contacted patients who were lost to follow-up for status updates after 6 months. Patients were presumed dead if they were not reachable by telephone and were withdrawn from the Taiwan National Health Insurance plan. Patient survival is the time from catheter implantation to death or last follow-up. Albumin (*n* = 15, 3.88%) and PG-SGA scores (*n* = 12, 3.11%) had missing data. After confirming a random missing pattern, we used multiple imputations (5 imputations) to replace these values.

Definitions:

Bacteremia: the presence of a positive pathogen (except contamination) in blood culture. Catheter-related bloodstream infection requires at least 1 of the following:

(i) Positive culture of the catheter tip or port reservoir associated with a positive peripheral blood culture with the same microorganism or the difference in time to positivity of blood culture drawn from the catheter versus that from a peripheral vein (positivity of the catheter blood sample was at least 2 h before that of the peripheral blood sample);
(ii) Local or general signs of infection, such as fever and chills, positive culture from the vascular port (catheter tip or the port reservoir), and regression of clinical signs of infection after port removal despite a negative peripheral blood culture [12]. We used clinical local signs such as local heat, erythema, and purulent discharge to diagnose pocket infection. Skin disruption and direct visualization of the port reservoir served for pocket exposure diagnosis.

### 2.2. Statistical Analysis

We conducted propensity score matching using logistic regression with unplanned early catheter removal as the dependent variable with a caliper width of 0.2 times the standard deviation of the propensity score without replacement to pair-match patients “with and without” unplanned early catheter removal. Due to the “no normal” distribution of data, we present continuous variables as median ± standard deviation and categorical variables as percentages. For the analysis of categorical variables, we used the Chi-square or Fisher’s exact test depending on the variable cell size, the Mann–Whitney U test for continuous variables, and the Kruskal–Wallis test for ordinary variables. We used standard definitions to convert continuous variables into categorical variables for analysis, as follows: leukopenia, white cell counts of less than 3.5 × 1000/μL; neutropenia, absolute neutrophil counts of less than 1.5 × 1000/μL; lymphopenia, absolute lymphocyte counts of less than 1.5 × 1000/μL; thrombocytopenia, platelet counts of less than 100 × 1000/μL; and hypoalbuminemia, albumin level of less than 3.5 g/dL. We classified body mass index (BMI) into three categories: underweight (BMI < 18.5), normal–overweight (BMI 18.5–30), and obesity (BMI > 30). Based on PG-SGA scores, we classified individuals into two categories for multivariate analysis: those with normal nutrition (scores 0–1) and those with malnutrition (scores 2–8). We use Cox proportional hazard analysis to estimate significance and relative risks with a 95% confidence interval (CI). A *p* value < 0.05 shows a statistically significant difference. We used commercial software, IBM SPSS Statistics for Windows, Version 29.0.2.0; Armonk, NY, USA: IBM Corp., for clinical data analysis.

### 2.3. Surgical Procedure

The surgical procedure was performed in the operation room as previously described [13]. Attending surgeons or senior surgical residents were responsible for all catheter implantation in the operation room under fluoroscopic control for the confirmation of the final catheter’s tip, using local anesthesia most of the time, and a single dose of prophylactic antibiotic with cephalosporin. Through a subclavian incision, the cephalic vein was selected for venotomy. If the cephalic vein was too small, tortuous, or unavailable, a subclavian or internal jugular vein puncture was used based on the surgeon’s discretion. The catheter was advanced from the venotomy site to the superior vena cava–right atrium junction under fluoroscopic guidance. After confirming the position of the catheter tip, the catheter was trimmed and connected to the injecting port. A small subcutaneous pocket was created, and the injection port was secured to the underlying pectoralis muscle fascia. The surgical incision was closed in layers, and the injecting port and catheter were filled with diluted heparin saline solution.

We used a non-coring Huber needle to inject through the implantable port, flushing the system after each injection or once every 4–12 weeks. We usually recommend removing the catheter after completing chemotherapy with at least a 24-month disease-free period, upon patient request, or if complications arise.

## 3. Results

**Figure 1:**
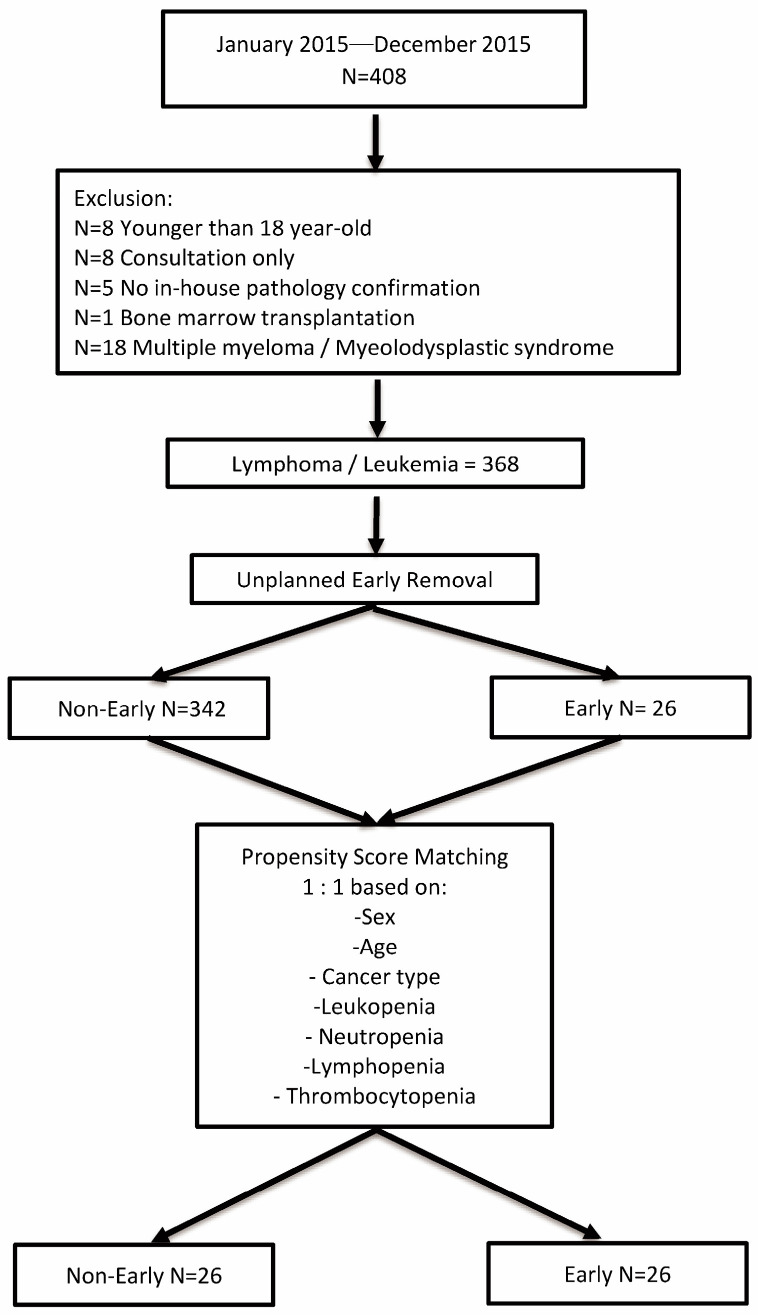
Patient recruitment and matching process.

We identified 408 patients with hematologic malignancies in our cancer registry. After excluding 40 patients, 368 remained in the final analysis, including 26 cases of unplanned early removal of implantable port catheters. With a total follow-up of 256,223 catheter days, the unplanned early implantable port catheter removal rate was 0.10/1000 catheter days. The reasons for catheter removal included bacteremia (*n* = 13), catheter-related bloodstream infection (*n* = 7), fever of unknown cause (*n* = 3), pocket infection (*n* = 1), pocket exposure (*n* = 1), and severe cardiac arrhythmia (*n* = 1). Univariate analysis of the unmatched cohort shows significant differences in cancer type, hemoglobin level, leukopenia, neutropenia, and thrombocytopenia. Figure 1 illustrates the propensity score matching process for patients with (early group) and without unplanned early catheter removal (non-early group).

In the unmatched cohort, significant differences were observed in cancer type (*p* < 0.001), hemoglobin level (*p* < 0.001), leukopenia (*p* = 0.003), neutropenia (*p* < 0.001), and thrombocytopenia. After propensity score matching, there were no statistically significant differences between early and non-early groups for age, sex, smoking history, body mass index, Charlson comorbidity score, creatinine, hypoalbuminemia, leukopenia, neutropenia, and lymphopenia. However, there were significant statistical differences for cancer type (*p* = 0.009), hemoglobin level (*p* < 0.001), and PG-SGA score (*p* = 0.001). Table 1 presents the unmatched and matched clinical characteristics.

Cox proportional hazard analysis showed no significant differences in hemoglobin levels (*p* = 0.831). The risk of unplanned early catheter removal was over four times higher in leukemia patients compared to lymphoma patients (HR 4.589, 95% CI 1.377–15.299, *p* = 0.013). According to the PG-SGA, patients with normal nutrition had a nearly 75% lower risk for unplanned early catheter removal (HR 0.258, 95% CI 0.116–0.575, *p* < 0.001). See Table 2 for details.

**Figure 2:**
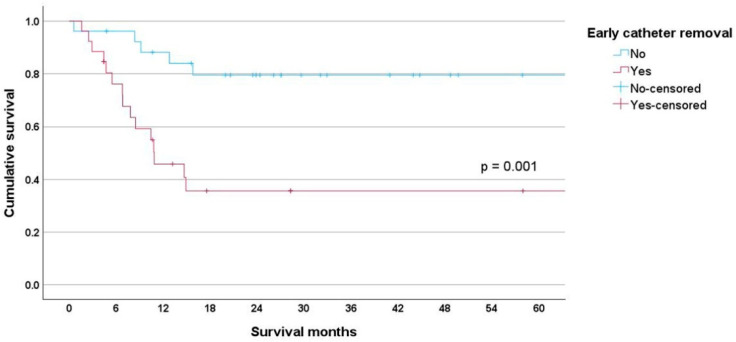
The mean survival was 60.78 months for the non-early group, decreasing to 33.80 months for the early group, *p* = 0.001.

Kaplan–Meier survival analysis showed significant differences between early and non-early groups (*p* = 0.001). The early group had a mean survival of 33.80 months (95% CI 19.292–48.309), while the non-early group had a mean survival of 60.78 months (95% CI 50.430–71.119) (Figure 2).

**Figure 3:**
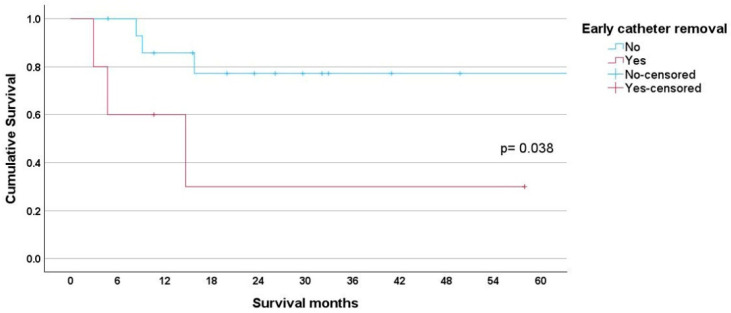
The mean survival for lymphoma patients in the early group was 23.32 months (95% CI 0.551–46.096) compared to 59.66 months (95% CI 45.364–73.953) for the non-early group, *p* = 0.038.

Early catheter removal was associated with reduced survival in both lymphoma and leukemia patients. For lymphoma patients, the early group had a mean survival of 23.32 months (95% CI 0.551–46.096) compared to 59.66 months (95% CI 45.364–73.953) for the non-early group, *p* = 0.038 (Figure 3).

**Figure 4:**
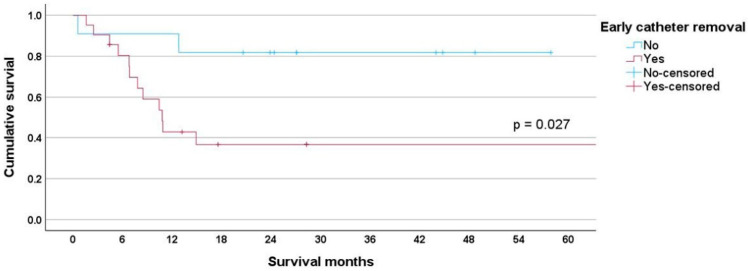
The mean survival for leukemia patients was 34.53 months (95% CI 18.477–50.586) for the early group compared to 48.48 months (95% CI 36.814–60.351) for the non-early group, *p* = 0.027.

For leukemia patients, the early group had a mean survival of 34.53 months (95% CI 18.477–50.586) compared to 48.48 months (95% CI 36.814–60.351) for the non-early group, *p* = 0.027 (Figure 4).

## 4. Discussion

In this propensity score matching study, we demonstrated that the disease, rather than the degree of cytopenia at the time of insertion, was the independent risk factor for unplanned early port catheter removal.

The treatment course of cancer patients, such as leukemia/lymphoma patients, often requires multiple rounds of chemotherapy, parenteral antibiotic therapy to treat infectious conditions, intravenous fluid infusion, blood product transfusion, and repeated blood draws. Reliable vascular access is crucial. While several central line catheters are available, totally implantable port catheters generally pose a lower complication risk [14,15]. However, infections and mechanical issues can lead to their early removal, which is problematic because of treatment interruption, prolonged hospital stays, and increased medical costs. Lymphoma and leukemia are two primary subsets of hematologic malignancies, often associated with varying degrees of cytopenia, thereby increasing the risk of infection. Leukopenia, notably its subset neutropenia, serves as an indicator of underlying immunosuppression. The association between neutropenia and port infection risk is unclear. Some studies reported a higher risk [7,8,16,17], while others found no association [18,19,20,21]. In the unmatched cohort, leukopenia (*p* = 0.003) and neutropenia (*p* < 0.001) were associated with unplanned early catheter removal. In contrast, in the matched cohort, neither leukopenia nor neutropenia increased the risk of unplanned early removal of implantable catheters (Table 1).

Platelets present immune function in addition to their essential role in the coagulation cascade. Platelets are recruited to infection sites, bind to pathogens, and prevent their dissemination [22,23]. Thrombocytopenia may result from decreased bone marrow production, increased destruction, splenic sequestration, or drug treatment effects. Thrombocytopenia is reported to be more common among patients with hematologic malignancies than among those with solid tumors. According to a recent Danish study [24], thrombocytopenia is present in 18% of patients with hematologic malignancies compared to only 4% of those with solid tumors. Among hematologic malignancies, leukemia presents with a higher prevalence of thrombocytopenia. Several studies found thrombocytopenia to be a risk factor for early port infection [13], mainly when associated with leukopenia or pancytopenia [17]. In this report on hematologic malignancies, which included patients with lymphoma and leukemia, thrombocytopenia was associated with unplanned early catheter removal in the unmatched cohort (*p* = 0.002). However, no such association was observed in the propensity-matched cohort (Table 1). Keulers et al. [25] also reported on the safety of port implantation in patients with thrombocytopenia.

Anemia is prevalent in lymphoma/leukemia patients. We found a median hemoglobin level of 9.80 ± 2.45 mg/dL in this study. Univariate analysis showed lower hemoglobin levels in early-group patients compared to the non-early group (9.15 ± 1.87 vs. 10.45 ± 2.68 mg/dL, *p* < 0.001). However, Cox proportional hazard analysis found no significant difference (HR 0.831, 95% CI 0.773–1.230, *p* = 0.831) (Table 2). This difference is clinically insignificant, as all patients were stable, and the procedure had low bleeding risk. Hemoglobin levels in neither group fell below the transfusion threshold of 7 mg/dL recommended by the Association for the Advancement of Blood and Biotherapies for stable hospitalized adults with hematologic and oncologic disorders [26].

Malnutrition in cancer patients can increase complications, reduce treatment effectiveness, lower survival rates, and increase healthcare costs [27]. Malnutrition can be present at presentation or result from cancer treatment. An estimated 40–80% of cancer patients experience malnutrition during their illness [28]. Its prevalence varies by tumor type, location, stage, and treatment [29]. The Patient-Generated Subjective Global Assessment (PG-SGA) is an established tool for nutritional screening, assessment, monitoring, and triaging adult oncology patients [11,30,31]. Malnutrition measured by PG-SGA score was previously unreported regarding port A outcomes. In the unmatched cohort, nearly 70% of patients were malnourished based on their PG-SGA. After matching, 35% were malnourished at diagnosis. There was a significant difference between early and non-early groups in univariate analysis (Table 1). Cox proportional hazard analysis found that patients with normal nutrition had a 75% lower risk of unplanned early catheter removal (HR 0.258, 95% CI 0.116–0.575, *p* < 0.001) (Table 2). Hypoalbuminemia is frequently used as a surrogate for malnutrition. Zhang et al. [5] previously reported the association of hypoalbuminemia (serum albumin < 3.5 mg/dL) and early port infection with a hazard ratio of 5.03. However, we did not find such a relationship.

In the literature, the association between cancer type and unplanned early catheter removal is conflicting. Groeger previously reported higher catheter-related infections for patients with leukemia compared to patients with lymphoma or myeloma [32], while other studies found no significant difference between patients with leukemia or lymphoma [5,13]. In this study of only lymphoma and leukemia patients, we identified cancer type as a significant risk factor for unplanned early catheter removal. The type of cancer was significantly associated with unplanned early catheter removal in both the unmatched cohort (*p* < 0.004) and the matched cohort (*p* = 0.003), as shown in Table 1. According to the Cox proportional hazard analysis, leukemia patients had a risk of unplanned early catheter removal that was over four times higher than that of lymphoma patients (HR 4.589, 95% CI 1.377–15.299, *p* = 0.013), as seen in Table 2. This difference could be attributed to varying cytopenia patterns observed between lymphoma and leukemia patients (Table 3). A higher proportion of leukemia patients exhibited anemia, leukopenia, neutropenia, and thrombocytopenia in comparison to lymphoma patients.

Patients with leukemia frequently present with anemia, leukopenia/neutropenia, and thrombocytopenia. While individual types of cytopenias were not associated with increased risk, their combination may potentially contribute to an increased risk in leukemia patients.

Unplanned early catheter removal disrupts treatment, increases costs, and negatively impacts patient survival. The mean survival was 60.78 months for the non-early group, decreasing to 33.80 months for the early group, *p* = 0.001 (Figure 2). This adverse survival affected both lymphoma and leukemia patients. For lymphoma patients, the mean survival for the early group was 23.32 months (95% CI 0.551–46.096) compared to 59.66 months (95% CI 45.364–73.953) for the non-early group (Figure 3). For leukemia patients, the mean survival was 34.53 months (95% CI 18.477–50.586) for the early group compared to 48.48 months (95% CI 36.814–60.351) for the non-early group (Figure 4).

We acknowledge the limitations of this study. The population consisted entirely of individuals of Chinese descent, which may limit the application of these results to other ethnic groups. Furthermore, this study’s small sample size, retrospective design, and lack of clinical information regarding post-chemotherapy cytopenia management are notable constraints. Despite these issues, the use of propensity score matching effectively mitigated bias from confounding variables, even with significant cytopenia differences at the time of catheter insertion between lymphoma and leukemia patients.

## 5. Conclusions

For patients with lymphoma and leukemia, malnutrition and the type of cancer, rather than independent cytopenia at the time of insertion, were identified as independent risk factors for unplanned early catheter removal, which can negatively impact patient survival. Further research is needed to determine effective prevention strategies for this condition.

## Figures and Tables

**Table 1:** Cohort and matched cohort demographic characteristics.

	Unmatched Cohort				Matched Cohort			
Parameter	All	Non-Early Group	Early Group	*p*-Value	All	Non-Early Group	Early Group	*p*-Value
Characteristics								
Number of patients n (%)	368 (100)	342 (92.90)	26 (7.10)		52 (100)	26 (50.00)	26 (50.00)	
Age (median + SD in years)	62.50 ± 15.47	63 ± 15.38	59.50 ± 16.05	0.542	57.50 ± 13.93	54.00 ± 11.63	59.50 ± 16.05	0.087
Sex n (%)				0.447				0.397
Female	144 (100)	132 (91.70)	12 (8.30)		21 (100)	9 (42.90)	12 (57.10)	
Male	224 (100)	210 (93.80)	14 (6.30)		31 (100)	17 (54.80)	14 (45.20)	
Smoking history n (%)				0.723				0.465
No	293 (100)	273 (93.20)	20 (6.80)		43 (100)	23 (53.50)	20 (46.50)	
Yes	75 (100)	69 (92.00)	6 (8.00)		9 (100)	3 (33.33)	6 (66.67)	
Cancer type				<0.001				0.004
Leukemia	133 (100)	112 (84.20)	21 (15.80)		32 (100)	11 (34.40)	21 (65.60)	
Lymphoma	235 (100)	230 (97.90)	5 (2.10)		20 (100)	15 (75.00)	5 (25.00)	
Body Mass Index n (%)				0.262				0.099
Underweight	16 (100)	15 (93.80)	1 (6.30)		2 (100)	1 (50.00)	1 (50.00)	
Normal-Overweight	324 (100)	303 (93.50)	21 (6.50)		46 (100)	25 (54.30)	21 (45.70)	
Obese	28 (100)	24 (85.70)	4 (14.30)		4 (100)	0 (0)	4 (100.00)	
Charlson comorbidity score (median + SD)	3.00 ± 1.96	3.00 ± 1.98	3.00 ± 1.77	0.504	2.5 ± 1.54	2.00 ± 1.16	3.00 ± 1.77	0.504
Creatinine (median + SD in mg/dL)	0.83 ± 0.56	0.82 ± 0.56	0.90 ± 0.63	0.451	0.83 ± 0.59	0.82 ± 0.56	0.90 ± 0.63	0.676
Hemoglobin (median + SD in g/dL)	10.90 ± 2.47	11.10 ± 2.47	9.15 ± 1.87	<0.001	9.80 ± 2.45	10.45 ± 2.68	9.15 ± 1.87	<0.001
Hypoalbuminemia				0.780				0.465
No	310 (100)	287 (92.60)	23 (7.40)		43 (100)	20 (46.50)	23 (53.50)	
Yes	58 (100)	55 (94.80)	3 (5.2)		9(100)	6 (66.70)	3 (33.33)	
Leukopenia n (%)				0.003				0.061
No	311 (1000	295 (94.90)	16 (5.10)		38 (100)	22 (57.90)	16 (42.10)	
Yes	57 (100)	47 (82.50)	10 (17.50)		14 (100)	4 (28.60)	10 (71.40)	
Neutropenia n (%)				<0.001				0.071
No	308 (100)	293 (95.10)	15 (4.90)		36 (100)	21 (58.30)	15 (41.70)	
Yes	60 (100)	49 (81.70)	11 (18.30)		16 (100)	5 (31.30)	11 (69.80)	
Lymphopenia n (%)				0.565				0.397
No	147 (100)	138 (93.90)	9 (6.10)		21 (100)	12 (57.10)	9 (42.90)	
Yes	221 (100)	204 (92.30)	17 (7.70)		31 (100)	14 (45.20)	17 (54.80)	
Thrombocytopenia n (%)				0.002				0.266
No	268 (100)	256 (95.50)	12 (4.50)		28 (100)	16 (57.10)	12 (42.90)	
Yes	100 (100)	86 (86.00)	14 (14.00)		24 (100)	10 (41.70)	14 (58.30)	
PG-SGA (Scored Patient Generated Subjective Global Assessment) n (%)								
Total PG-SGA Score				0.162				0.001
Normal nutrition	111 (100)	100 (90.10)	11 (9.90)		34 (100)	23 (67.60)	11 (32.40)	
Malnutrition	257 (100)	242 (94.20)	15 (5.80)		18 (100)	3 (16.70)	15 (83.30)	

**Table 2:** Adjusted Cox proportional hazard analysis for unplanned early catheter removal.

Early Unplanned Catheter Removal			
Parameter	Adjusted Hazard Ratio	95% CI	*p* Value
Hemoglobin	0.831	0.773–1.230	0.831
Diagnosis			
Lymphoma	Ref		
Leukemia	4.589	1.377–15.299	0.013
PG-SGA			
Malnutrition	Ref		
Normal nutrition	0.258	0.116–0.575	<0.001
PG-SGA: Scored Patient Generated Subjective Global Assessment			
Abbreviations: CI, confidence interval; Ref, reference			

**Table 3:** Matched demographic characteristics for lymphoma and leukemia patients.

Parameter				
Characteristics	All	Lymphoma	Leukemia	*p* Value
Number of patients n (%)	52 (100)	23 (44.20)	29 (55.80)	
Age (median + SD in years)	57.50 ± 13.93	58.00 ± 14.98	53.00 ± 113.46	0.706
Sex n (%)				0.964
Female	21 (100)	8 (38.10)	13 (61.90)	
Male	31 (100)	12 (38.70)	19 (61.30)	
Smoking history n (%)				0.719
No	43 (100)	16 (37.20)	27 (62.80)	
Yes	9 (100)	4 (44.40)	5 (55.60)	
Body Mass Index n (%)				0.892
Underweight	2 (100)	0 (0)	2 (100.00)	
Normal–Overweight	46 (100)	18 (39.10)	28 (60.90)	
Obese	4 (100)	0 (0)	4 (100.00)	
Charlson comorbidity score (median + SD)	2.50 ± 1.54	2.50 ± 1.36	2.50 ± 1.66	0.808
Creatinine (median + SD in mg/dL)	0.83 ± 0.59	0.82 ± 0.90	0.88 ± 0.24	0.498
Hemoglobin (median + SD in g/dL)	9.80 ± 2.45	12.25 ± 2.10	8.65 ± 1.69	<0.001
Hypoalbuminemia				1.000
No	43 (100)	17 (39.50)	26 (60.50)	
Yes	9 (100)	3 (33.33)	6 (66.67)	
Leukopenia n (%)				0.005
No	38 (100)	19 (50.00)	19 (50.00)	
Yes	14 (100)	1 (7.10)	13 (92.90)	
Neutropenia n (%)				0.001
No	36 (100)	19 (52.80)	17 (47.20)	
Yes	16 (100)	1 (6.30)	15 (93.80)	
Lymphopenia n (%)				0.532
No	21 (100)	7 (33.30)	14 (66.70)	
Yes	31 (100)	13 (41.90)	18 (58.10)	
Thrombocytopenia n (%)				<0.001
No	28 (100)	18 (64.30)	10 (35.70)	
Yes	24 (100)	2 (8.30)	22 (91.70)	
PG-SGA (Scored Patient Generated Subjective Global Assessment) n (%)				0.580
Normal nutrition	34(100)	14 (41.20)	20 (58.80)	
Malnutrition	18 (100)	6 (33.33)	12 (66.70)	
Early catheter removal n (%)				0.009
No	26 (100)	15 (57.70)	11 (42.30)	
Yes	26 (100)	5 (19.20)	21 (80.80)	

## Data Availability

The data presented are available upon request to the corresponding author.
